# Sub-symptom threshold aerobic exercise for patients with persisting post-concussion symptoms and exercise intolerance after mild traumatic brain injury – a study protocol with a nested feasibility study for a randomized controlled trial

**DOI:** 10.1186/s12883-023-03221-7

**Published:** 2023-05-03

**Authors:** Lars-Johan V. Valaas, Helene L. Soberg, Mari S. Rasmussen, Sophie E. Steenstrup, Nada Andelic, Ingerid Kleffelgård

**Affiliations:** 1 Department of Physical Medicine and Rehabilitation, Oslo University Hospital, Oslo, Norway; 2 Center for Habilitation and Rehabilitation Models and Services (CHARM), Faculty of Medicine, Institute of Health and Society, University of Oslo, Oslo, Norway; 3 Department of Rehabilitation Science and Health Technology, Faculty of Health Sciences, Oslo Metropolitan University, Oslo, Norway

**Keywords:** mTBI, Exercise intolerance, Buffalo Concussion Treadmill Test, Sub-symptom threshold aerobic exercise, Persistent post-concussion symptoms

## Abstract

**Background:**

Persistent post-concussion symptoms (PPCS) affect between 34 and 46% after a mild traumatic brain injury (mTBI). Many also experience exercise intolerance. Sub-symptom threshold aerobic exercise, SSTAE (exercise at an intensity level that does not increase symptoms) is proposed as a treatment to both reduce the symptom burden and increase the exercise tolerance after the injury. It is unclear if this also applies in a more chronic phase after mTBI.

**Main purpose:**

The main purpose of this study is to evaluate whether SSTAE in addition to ordinary rehabilitation will lead to clinically meaningful improvement of symptom burden, normalize exercise tolerance, increase physical activity, improve health-related quality of life, and reduce patient-specific activity limitations compared to a control group that only receives ordinary rehabilitation.

**Design:**

Randomized, controlled, single-blind parallel-group study with three measurement times; T0 at baseline, T1 after the intervention and T2 six months after T1.

**Methods:**

Patients between the ages of 18 and 60 with exercise intolerance and persistent PPCS (> 3 months) will be recruited to the study and randomized to two groups. All patients will receive follow-up at the outpatient TBI clinic. The intervention group will in addition receive SSTAE for 12 weeks with exercise diaries and a retest every 3 weeks for optimal dosage and progression. The Rivermead post-concussion symptoms questionnaire will be the main outcome measure. The secondary outcome measure will be a test of exercise tolerance—the Buffalo Concussion Treadmill Test. Other outcome measures include the patient-specific functional scale that measures patient-specific activity limitations, as well as outcome measures for diagnosis-specific health-related quality of life, anxiety and depression, specific symptoms such as dizziness, headache and fatigue, and physical activity.

**Discussion:**

This study will add knowledge about the effect of SSTAE and whether it should be implemented in rehabilitation for the adult population with persistent PPCS after mTBI. The nested feasibility trial showed that the SSTAE intervention was safe and that the study procedures and delivery of the intervention overall were feasible. However, minor amendments to the study protocol were made prior to the commencement of the RCT.

**Trial registration:**

Clinical Trials.gov, NCT05086419. Registered on September 5th, 2021.

**Supplementary Information:**

The online version contains supplementary material available at 10.1186/s12883-023-03221-7.

## Background

Exercise, as a rehabilitation intervention for patients with persistent symptoms after mild traumatic brain injury (mTBI), will be explored in this randomized controlled trial (RCT). Traumatic brain injury (TBI) is a complex injury defined as a change in brain function or other signs of brain pathology caused by an external force [1]. Between 80–90% are mTBI, with an annual incidence in Norway of 302 pr. 100,000 [2].

The most common causes of mTBI injuries are falls, traffic, sports related accidents and violence [3–5]. Many recover well during the first three months after the injury, but between 34 and 46% report cognitive, psychological, and physical persistent post-concussion symptoms (PPCS) at three and six months [6]. More specifically, headache, dizziness, fatigue, emotional distress, problems with memory, concentration and slowed thinking are common PPCS [6, 7]. In addition, exercise intolerance, PPCS exacerbation during physical activity, is challenging to manage. Because many patients with mTBI are young and of working age [2], it is important to develop effective rehabilitation interventions.

Traditionally, mTBI management has consisted of advice on physical and cognitive rest until the PPCS wanes, and then gradually resume activities [7–9]. However, this approach is questioned [7] as studies show no effect of rest on PPCS after mTBI [10–12]. Studies rather suggest that cognitive and physical rest beyond the first few days contributes to persistent PPCS [8, 11]. PPCS might also reduce activity and participation, and is associated with reduced health-related quality of life [13]. Hence, a more physically active approach in rehabilitation after mTBI should be explored [8, 9, 14].

Studies indicate that both central and systemic physiological dysfunction can contribute to exercise intolerance after mTBI [9, 14]. Exercise intolerance can be understood as reduced ability to be physically active/exercise at the expected level according to age and physical condition due to exacerbation of symptoms [14–16]. Research suggests that metabolic and physiological changes in the acute phase after mTBI may lead to autonomic dysfunction and abnormal control of cerebral blood flow. This can cause increased symptoms (e.g., headache and dizziness) with exertion and lead to exercise intolerance [9, 14]. The risk of developing exercise intolerance with symptom exacerbation during physical activities is believed to increase 3–18 times after mTBI compared to healthy controls [14, 15]. However, it is unclear whether this also applies in a more persistent phase after mTBI.

Aerobic exercise at an intensity level that does not increase symptoms (sub-symptom threshold aerobic exercise, SSTAE) has received increasing focus in rehabilitation of patients with PPCS after mTBI. Recent studies suggest that SSTAE is safe, reduces PPCS, and may contribute to fewer activity limitations and increased participation [7, 17, 18]. However, studies utilizing an adapted exercise program have mainly studied young athletes [7, 19] or general instructions about physical activity to the mTBI adult population in the acute phase as mean to prevent PPCS [20]. The Buffalo Concussion Treadmill Test (BCTT) is a safe and validated treadmill test that has been developed to evaluate exercise intolerance after mTBI [8, 9, 21, 22]. The BCTT is designed to identify symptom exacerbation thresholds while SSTAE may be utilized to guide the exercise intensity based on the symptom threshold, i.e., sub-symptom exercising [8, 9, 14, 15, 17, 21–23].

To our knowledge, no larger clinical trials determining the effects of SSTAE in patients suffering from exercise intolerance and PPCS after mTBI in the general adult population have currently been concluded [7, 24]. Hence, there is a lack of evidence-based knowledge about the effect of SSTAE in adult patients in a more chronic phase after the injury. Few larger studies with control groups have made it difficult to draw good evidence-based guidelines for clinical practice [7]. Thus, there is a need for randomised clinical trials (RCT) testing the effect of SSTAE in the adult population with PPCS. The main purpose of this study is to explore the effect of SSTAE on PPCS and exercise intolerance in patients with mTBI compared with a control group receiving treatment as usual (TAU) only.

## Objectives

The aim of this study is to explore whether SSTAE, in addition to TAU, will reduce symptom burden and normalize exercise tolerance compared with a control group that only receives TAU.

Further, we aim to evaluate the effect of SSTAE on changes in symptoms such as headache, fatigue, anxiety, and depression, and to register changes in physical activity, health-related quality of life and patient-specific activity limitations.

Our specific hypotheses are:

- H1: The SSTAE intervention will result in a greater reduction in PPCS symptom burden compared with TAU only.
- H2: The SSTAE intervention will result in a greater normalisation of exercise tolerance compared with TAU only.
- H3: The SSTAE intervention will result in greater improvement in headaches, fatigue, anxiety, depression, physical activity, and health-related quality of life compared with TAU only.
- H4: The SSTAE intervention will result in greater improvement in patient-specific activity limitations compared with TAU only.

In accordance with the New Medical Research Council guidelines [25] we conducted a feasibility study as a preparatory step before initiating the RCT. The feasibility study is nested in this protocol article. The aim of the feasibility study was to evaluate the intervention arm using predefined success criteria shown in Table 2.

### Trial design

This is as a randomized, controlled, single-blind clinical trial with two parallel groups allocated with a 1:1 ratio, and a primary endpoint of difference in PPCS burden at 12 weeks. See Table 1 for Standard Protocol Items: Recommendations for Interventional Trials (SPIRIT) [26, 27].

**Table 1:** Standard Protocol Items: Recommendation for interventional Trials (SPIRIT) of the current randomized controlled trial

		**STUDY PERIOD**				
	**Enrolment**	**Baseline test**	**Allocation**	***Intervention***	**Assessments**	
**TIMEPOINT**		***T***_***0***_			***T***_***1***_*** (12 weeks)***	***T***_***2***_*** (6 months)***
**ENROLMENT:**						
**Eligibility screen**	X					
**Informed consent**	X					
**Allocation**			X			
**INTERVENTIONS:**						
***SSTAE group***				X		
***TAU group***				X		
**ASSESSMENTS:**						
**Primary outcome measures:** ***Post-concussion symptoms – RPQ***		X			X	X
***Secondary outcome measure:*** **Exercise intolerance – BCTT**		X			X	X
***Other outcomes***		X			X	X

*Abbreviation*: *SSTAE* Sub-symptom threshold aerobic exercise, *TAU* Treatment as usual, *RPQ* Rivermead Post-concussion symptoms questionnaire, *BCTT* Buffalo Concussion Treadmill Test

Patients referred to the TBI outpatient clinic will be screened for eligibility by the treating physician and invited to participate in the study. Informed consent will be collected and baseline assessment (T0), including patient reported outcome measures and test of exercise tolerance, will be conducted (Table 3). After the baseline assessment, participants will be randomized to SSTAE intervention or TAU group. Further, follow-up testing will be carried out after the intervention period at 12 weeks (T1) and 6 months (T2) after the baseline assessment.

The nested feasibility study was a non-randomized, feasibility study of the intervention arm of the RCT, and included baseline assessment and follow-up after 12 weeks of SSTAE. According to the predefined success criteria, we assessed the recruitment process, retention rates, outcome measures, and adherence to the intervention. A convenience sample including 11 patients with mTBI and self- reported exercise intolerance was recruited to the feasibility study from October – November 2021.

## Methods: patients, interventions, and outcomes

### Study setting

The clinical trial will be carried out at the TBI outpatient clinic at the Department of Physical Medicine and Rehabilitation, Oslo University Hospital (OUH), the trauma referral centre in South-East Norway [28]. Patients are referred to the TBI outpatient clinic from the Emergency department, Neurosurgical department, and from general practitioners. Assessments and clinical testing will be performed in an exercise laboratory at the TBI outpatient clinic. The exercise program will be conducted in the patients’ home setting. The feasibility study was carried out within the same study setting.

### Eligibility criteria

The following inclusion and exclusion criteria will be applied in this trial:

- Inclusion criteria: Patients 18–60 years diagnosed with mTBI as defined by the World Health Organization Collaborating Center Task Force [29] with persistent PPCS 3 – 24 months post injury and reduced tolerance of physical activity, defined as self-reported exacerbation of PPCS during physical activity and exercise.
- Exclusion criteria: Patients with neurological or severe psychiatric conditions documented in the medical record, cardiovascular disease that contraindicates exercise testing [30], extremity injuries that prevent physical exercise, ongoing substance abuse, and insufficient understanding of Norwegian to follow instructions or fill in forms will be excluded before baseline testing. Additionally, patients with no self-reported exacerbation of PPCS during the BCTT will be excluded.
- In the feasibility study, patients who did not fulfil the stop criteria for the BCTT but experienced symptom exacerbation within 24 h after the test were also included.

### Consent

All patients will receive written and oral information about the study from the treating physician or physiotherapist prior to the baseline assessment and provide their written informed consent. The procedure for obtaining informed consent in the feasibility study was consistent with the current protocol.

## Interventions

### The Buffalo Concussion Treadmill Test—BCTT

The BCTT is the secondary outcome in this trial, but it is also an essential part of the SSTAE intervention and will therefore be described here. The BCTT is a standardized incremental treadmill test designed to identify exercise intolerance and establish symptom thresholds in mTBI patients [17, 21, 22, 31]. The patient walks at a brisk pace on a treadmill (5.2 – 5.8 kph), as incline is increased by 1% each minute. If 15% incline is reached, the speed is increased by 0.3 km/h each minute. The PPCS rated on a 0–10 (best–worst) numerical rating scale (NRS), perceived exertion on Borg Ratings of Perceived Exertion (RPE) 6–20 (lowest to highest) [32], and heart rate (HR) are recorded at the test’s baseline and each minute. The BCTT is terminated when one of three criteria is reached: 1) ≥ 3 points increase in PPCS on the NRS; 2) Borg RPE ≥ 18; 3) 90% of age estimated max HR calculated by (211–0.65 × age) [33]. The first criterium is interpreted as exercise intolerance, and the associated HR is the symptom threshold. The test is considered negative for exercise intolerance if terminated by the intensity criteria, and these patients will be excluded. In the feasibility study, the BCTT was administrated with stop criteria 1 and 2. The third stop criterion was added for the planned RCT to ensure that the participants did not exceed 90% of their estimated HRmax level. We also revised the symptom registration from registration of individual symptoms to registration of an overall PPCS score on the NRS to ensure a total symptom exacerbation of NRS ≥ 3 points.

### Sub-symptom threshold aerobic exercises (SSTAE)

The SSTAE intervention comprises consultations, BCTT testing and a personalized exercise program. The patients are instructed to perform aerobic exercise in their preferred mode, at an intensity of 80–90% of the symptom threshold, as identified during the BCTT, i.e., at the sub-symptom threshold [22, 23]. Each session follows this plan: 5–10 min warm up, 20 min aerobic exercise at sub-symptom threshold intensity, followed by 5–10 min of active cool down. The intensity of the SSTAE is monitored using a HR monitor and/or the Borg RPE scale. If symptoms exacerbate during an exercise session with ≥ 3 NRS, or perpetuate the following days, the patients are advised to reduce the HR with 5–10 beats/minute to reduce the PPCS. Exercise frequency is jointly decided by patient and therapist, from 3 to 5 sessions per week, based on exercise intensity, symptom burden and other external loads. The patients are encouraged to contact physiotherapist (LJVV) by phone for advice and reassurance if necessary. If needed, patients will be referred to a physiotherapist in primary health care service for exercise adherence.

To ensure appropriate exercise dosing, adherence and progression, the patients are offered two guided treadmill sessions during the first two weeks of the intervention in addition to a new BCTT at week 3, 6, 9 prior to T1 after 12 weeks. Compliance with the SSTAE will be recorded using an exercise diary and constitute the basis for the consultations focusing on supporting, motivating, and reassuring the patients. The control group is followed up with assessments and BCTT at baseline (T0), after 12 weeks (T1) and six months (T2) only.

In the feasibility study, the described SSTAE intervention arm was implemented and tested on all included patients.

### Treatment as usual

All patients included in the study will receive TAU at the TBI outpatient clinic. The TAU comprises individual contacts and an educational group provided by a multidisciplinary team. The specific treatment each participant receives varies according to individual needs. A specialist in physical medicine and rehabilitation addresses physical problems related to the injury, while a neuropsychologist addresses psychological or cognitive complaints. An occupational therapist helps the patients structure their day and a social worker advise patients on issues relating to work, legal rights, and benefits. A physical therapist addresses vestibular symptoms and physical activity. In addition, the educational group entails meeting 2 h once a week over a period of 4 weeks and addresses general information about mild-to-moderate TBI, common symptoms and problems in daily life, and advice regarding how to manage these [34]. The TAU includes general advice on physical activity based on recommendations from the Norwegian Directorate of Health, but not specific guidance on SSTAE and help with exercise dose.

#### Feasibility of the recruitment process and the intervention arm of the RCT

The nested feasibility study in this protocol article was based on pre-defined objectives with success criteria grounded on recommendations from literature [25, 35] and experience from previous clinical trials at the department of Physical Medicine and Rehabilitation. The procedures described in the protocol for T0 at baseline to T1 at 12 weeks were followed.

Eighteen patients were consecutively assessed for eligibility, five declined participation, leaving 13 for the baseline assessment. Two were excluded due to negative BCTT or aberrant HR during BCTT, leaving 11 patients, eight women and three men, with exercise intolerance included in the feasibility study. Two patients were lost to follow up during the intervention.

The mean age of the 11 patients completing the baseline assessment with exercise intolerance was 36.9 (SD 11.4) years. All had Glasgow Coma Scale score of 15 at the time of injury; one of them had intracranial injury on CT scan. Mean time since injury was 5.1 (SD 2.5) months. Five patients sustained their injuries from falls, two by sports-related activities, one from violence and three from miscellaneous situations. The predefined success criteria and the results from the feasibility study are presented in Table 2.

**Table 2:** Study objectives, success criteria and results of the feasibility study

Predefined Success Criteria	Results
**Recruitment process**	
> 50% of the eligible patients willing to participate in the study	18 patients screened eligible. 13 accepted invitation. Consent rate was 72%
**Outcome assessments**	
Appropriate selection of PROMs	Removal of Dizziness Handicap Inventory. Replaced QOLIBRI with QOLIBRI-Overall Scale. Added Problematic Experience of Therapy Scale.
Max 40 min completing the PROMs	12 of 13 patients (92%) completed the PROMs in < 40 min
< 10% missing PROM items	0.3% of total items missing
≥ 80% of patients have exercise intolerance confirmed with the BCTT	12 of 13 patients (92%) had exercise intolerance according to BCTT. One patient had normal test but increased symptoms in the next 24 h. 11 patients with mean age of 36.9 (SD 11.4) were included and were invited for retest
Assessment of primary and secondary outcome for RCT 1. Symptom burden: Rivermead Post-Concussion Symptoms (RPQ) 2. Test for exercise intolerance: Buffalo Concussion Treadmill Test (BCTT)	9 patients received the intervention and were retested The primary outcome measuring symptom burden (RPQ) captured reduction in PPCS.BCTT showed increased exercise tolerance. See Supplementary file for figure
**Retention rates**	
< 20% drop out	2 of 11 included patients (18%) were lost to follow-up
**Adherence to the intervention**	
> 67% attendance rate of introductory therapy sessions of the intervention	Attendance rate of 90%
**Patients adhere to min. 3 weekly prescribed unsupervised exercise sessions**	75% performed exercise according to prescription
> 67% of the patients maintains exercise diary	8 of 9 patients (89%) maintained exercise diary
**Ancillary non-predefined objectives**	
Patients that report increased PPCS next 24-h after test was included	Patients requires 3-point symptom increase during BCTT to be included
Stop criteria for the BCTT: The patients continued the test until voluntary exhaustion or Borg RPE > 18	Stop criteria BCTT added: 90% estimated max HR (211–0.64*age)
Multiple NRS score per symptom	Overall PPCS NRS, that includes all symptoms that patients relate to PPCS

*PROM* Patient Reported Outcome Measure, *DHI *Dizziness Handicap Inventory, *QOLIBRI* Quality of life after traumatic brain injury, *PPCS Persistent* Post Concussion Symptoms, *NRS* Numerical Rating Scale, *BCTT* Buffalo Concussion Treadmill Test

The results for the primary and secondary outcomes in the feasibility study are presented in more detail in the Supplementary file.

### Outcomes

The primary outcome is symptom burden measured with the Rivermead Post-Concussion Symptoms Questionnaire (RPQ). The secondary outcome is exercise intolerance measured with the BCTT. Other outcomes include headache symptoms and severity, fatigue, anxiety, psychological distress, depressive symptoms, estimates of physical activity, health-related quality of life and activity limitations, and adherence to the intervention. The selected outcome measures have satisfactory psychometric properties. The outcomes with references are displayed in Table 3. The outcomes will be administered at baseline (prior to group allocation), at 12 weeks and 6 months after baseline and the order of administration will be standardized. Adherence to the intervention will only be measured in the intervention group. In the feasibility study the same Patient Reported Outcome Measures (PROMs) were administrated with a few changes in the RCT (see Table 3).

**Table 3:** Outcome measures

**Outcome**	**Outcome Measure**	**Feasibility study**	**RCT**
Primary outcome			
Symptom burden	Rivermead Post-concussion Symptoms Questionnaire (RPQ) [36, 37]	x	x
Secondary outcome			
Exercise intolerance	Buffalo Concussion Treadmill Test (BCTT) [22, 23]	x	x
Other outcomes			
Headache symptoms and severity	Headache Impact Test (HIT-6) [38]	x	x
Fatigue	Fatigue Severity Scale (FSS)) [39]	x	x
Anxiety	Generalized Anxiety Disorder Scale (GAD-7) [40]	x	x
Depressive symptoms	Patient Health Questionnaire (PHQ-9) [41, 42]	x	x
Estimates of physical activity	International physical activity questionnaire (IPAQ) [43]	x	x
Health-related quality of life	Quality of Life after Brain Injury (QOLIBRI) [44]	x	
Health-related quality of life	Quality of Life after Brain Injury Overall Scale (QOLIBRI-OS) [45]		x
Adherence to the intervention	The Problematic Experience of Therapy scale (PETS) [46]		x
Dizziness	Dizziness Handicap Inventory (DHI) [47]	x	
Activity limitations	Patient-specific functional scale (PSFS) [48, 49]	x	x

#### Primary outcome

The RPQ [36] is a 16 items questionnaire about post-concussion symptoms rated on a 5-point scale from 0–4 where 0 = not experienced to 4 = severe problem. The scale range is 0–64 points (best–worst). The items cover physical symptoms (headache, dizziness, nausea, noise sensitivity, sleep disturbance, vision, and light sensitivity) and emotional/cognitive symptoms (fatigue, psychological distress, irritability, frustration, memory, concentration, speed of thinking, restlessness). The primary endpoint is the difference in change of symptom burden on the RPQ between the SSTAE and control groups from baseline to 12 weeks, with a follow up at 6 months after baseline.

#### Secondary outcome

The BCTT is described in detail above under the intervention. Changes in BCTT as reached percentage of estimated HRmax and changes in duration of the test from baseline (T0) to 12 weeks (T1), is the secondary endpoint.

In addition to outcome measures displayed in Table 3, demographic (age, sex, level of education, marital status, occupation, and sick leave status) and injury related variables (GCS, imaging finding, loss of consciousness (LOC), post-traumatic amnesia (PTA) and cause of injury) will be registered. Treatments outside of TAU initiated by the hospital or the patients themselves during the study period will be registered.

### Participant timeline

**Fig. 1:**
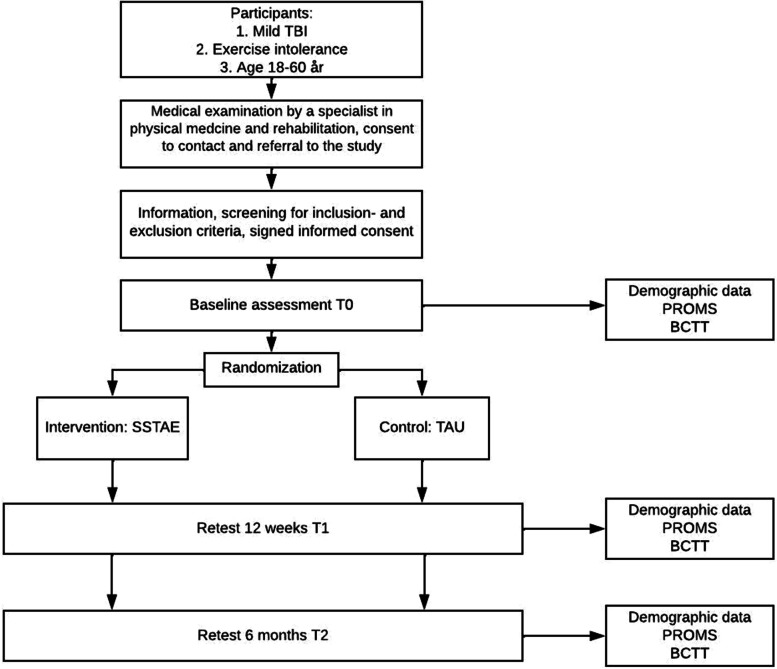
Flowchart of the RCT. PROMS: Patient Reported Outcome Measures; BCTT: Buffalo Concussion Treadmill Tes; SSTAE: Sub symptom threshold aerobic exercise; TAU: Treatment as usual

A study flowchart is provided in Fig. 1.

### Sample size

Based on RPQ and results from a pilot study at OUH (53), a power calculation was conducted with a SD of 8.7 and a mean group difference of 5.0 points on the RPQ. Given an 80% power and an alpha of 0.05, 96 participants should be included to find significant improvement. With an anticipated dropout rate of 20%, we will recruit 120 participants for this trial (60 in each group). No interim analysis is planned to change sample size during the recruitment period.

### Recruitment

Potential participants will be recruited from the TBI outpatient clinic at OUH. They will be informed about the study, screened for inclusion and exclusion criteria, and invited to participate by the treating physician or rehabilitation professionals. Eligibility will be confirmed at the baseline assessment prior to randomization. Approximately 270 patients between 18 – 60 years with mTBI are treated at the clinic per year. About 30% will meet the inclusion criteria, meaning approx. 80 patients pr. year will be qualified. If necessary, potential participants will be recruited from the TBI outpatient clinic at Sunnaas Rehabilitation Hospital to reach the target sample size.

### Assignment of interventions: allocation

#### Sequence generation

Patients will be randomized in a 1:1 ratio to the intervention or control group. To ensure allocation concealment, a computer-generated list with randomised variable block sizes will be prepared by an independent statistician. Sequentially numbered, opaque, sealed envelopes containing the allocation will be prepared by the senior researcher (HLS) and drawn consecutively after the baseline assessment.

#### Concealment

The allocation sequence list will be stored by a senior researcher not involved in the execution of the intervention. The therapist (LJVV) assigning the participants to the randomization, and the blinded assessors do not have access to this list.

#### Implementation

Eligible patients will be identified by the treating physician or rehabilitation professionals at the TBI outpatient clinic at OUH. The therapist delivering the intervention receives information about the potential eligible patients and contacts them by phone for further information and for scheduling the baseline assessment. The therapist will assign a study ID number to each participant scheduled for baseline assessment. Subsequently, when included in the study, the patients will be assigned a randomization number that is different from the study ID number. Only the senior researcher (HLS) will have access to the computer-generated list with randomization numbers.

### Blinding

Blinding of the patients or therapist to the treatment allocation is not possible. However, the randomisation occurs after baseline testing (T0), and the follow-up tests at T1 and T2 will be performed by an assessor blinded to the group allocation. The patients will be instructed not to disclose their group allocation. For the statistical analyses, dummy id-numbers will be used, and an independent statistician, blinded for the group allocation, will be responsible for the primary analyses of the data.

### Data collection, management, and analysis

#### Plans for assessment and collection of outcomes

The outcome assessors will be trained in the administration of all outcome measures. The data collection will occur at the three time points, baseline (T0), and 12 weeks (T1) and six months (T2) after baseline. The PROMs will be filled out and collected before the interview-based questionnaire and the BCTT. The PROMs will be checked for any missing items and if required, be completed by the patient.

#### Plans to promote participant retention and complete follow-up

To ensure adherence to the planned timing of the retests and follow-ups, one assigned researcher (LJVV) will administer all patient appointments.

To minimize control group discontent of not being allocated to the SSTAE intervention and potential loss to follow-ups, the control group will receive information about and be offered guidance in SSTAE after T2 if interested.

### Data management

All data material will be assigned an ID number. Only the project researchers will have access to list linking participants names to ID numbers. The de-identified documents will be stored according to national regulations. The de-identified documents will be transferred to an electronic data file on a research server at OUH in accordance with the Norwegian Data Protection Law. Only researchers that are actively contributing to statistical analyses and publication will have access to the final data set. Data entry quality will be ensured through initial exploratory analyses.

### Statistical methods

Statistical analyses will be performed in collaboration with an independent statistician at Oslo Centre for Biostatistics and Epidemiology at OUH. Updated IBM SPSS and RStudio software will be used for the statistical analysis.

#### Primary and secondary outcomes

Descriptive statistics will be used on socio-demographic and injury-related data. To analyze whether the intervention has a positive additional effect on symptom burden and exercise intolerance compared to the control group, a mixed-model analysis will be used. In this analysis, time (T0-T2) will be used as a «repeated-measures factor» and comparison between the intervention and the control group as a «between-group factor». The linear mixed model analyses will give estimated mean values with 95% confidence intervals for all time points (T0-T2) for each group. Estimates of mean between group changes from T0 to T1 and T2 will be provided. To establish treatment effect, the analysis of primary interest is a time x group interaction in the direction of the intervention group improving above the levels of the control group at T1. A significance level of 0.05 will be applied.

To reduce the risk of dropout bias, the analyzes will be performed on an intention-to-treat basis.

By including all participants in the analysis in the group they were randomized to.

The same mixed-model analyses will be performed for the PROMs described in our third hypothesis (H3).

#### Methods for any additional analyses (subgroups and adjusted analyses

The baseline population, including patients that are excluded due to a negative BCTT, will be described and analysed. Group differences between patients with and without a negative BCTT will be explored. Factors of PPCS and exercise intolerance at baseline will be analysed with multivariable regression analyses. Adherence to the intervention and changes on the BCTT will be described in the intervention group.

#### Methods to handle missing data

Missing data will be handled by questionnaire specific imputation, last observation carried forward and multiple imputation techniques. In the mixed-model analyses missing data will be handled by the analysis using the maximal likelihood approach under the assumption of missing at random.

### Data monitoring

#### Composition of the data monitoring committee and its role and reporting structure

An external data monitoring committee was considered unnecessary due to the small sample size and a relatively short timeframe of the intervention and follow-ups. To ensure adherence to the protocol, the date and time of all assessments and follow-ups are documented in both the intervention and control groups.

### Adverse event reporting and harms

Any harms and/or serious adverse events will be registered and reported in future publications.

### Frequency and procedures for auditing trial conduct

Not applicable.

### Ethics and dissemination

#### Research ethics approval

The study is approved by the regional ethical committee for Medical and Health Research Ethics (#256,109) and the OUH Data Protection Officer/Norwegian Center for Research Data. The project will be conducted according to the Helsinki declaration. The planned RCT and the feasibility study are registered at clinicaltrial.gov #NCT05086419.

### Confidentiality

Access to the list that can re-identify the patients will be restricted to a senior researcher (HLS).

Participant information will be handled by health-care professionals adhering to Norwegian law on confidentiality.

### Declaration of interests

No financial or other competing interests.

### Ancillary and post-trial care

All patients are covered by the Norwegian System of Patient Injury Compensation.

### Protocol amendments

Important amendments to the protocol will be approved by the regional Committee for Medical and Health Research Ethics and reported to the Data protection office at OUH. Amendments will be made to the clinicaltrials.gov registry.

### Dissemination policy

Results from this project will be disseminated through presentations at national and international conferences and published articles. It is expected that the study will result in 3–5 articles published in internationally recognized peer reviewed journals in the fields of physiotherapy, exercise physiology, sports medicine, and rehabilitation. The results will also be shared with user organizations.

The Consolidated Standards of Reporting Trials (CONSORT) will be applied to facilitate transparency and critical appraisal of the trial. Authorship criteria will adhere to the recommendations from the International Committee of Medical Journal Editors (ICMJE).

The project has a plan for user participation which is in line with the OUH research strategy. The goal is to ensure that users' perspectives, needs, and experiences are reflected in the project.

## Discussion

This RCT will contribute to increased knowledge about the effect of SSTAE on persistent PPCS and exercise intolerance after mTBI. If proven effective, the trial will support the implementation of SSTAE as part of the rehabilitation for this patient group. In particular, the trial will provide knowledge about the generalizability of the intervention to the adult population with persistent PPCS after mTBI, in addition to replicating the effectiveness of the SSTAE found in other studies mainly on young athletes. Furthermore, the trial will have the potential to expand our knowledge about how reduced symptom burden through SSTAE may influence quality of life, and activity limitations in this group of patients.

The nested feasibility trial showed that BCTT and the SSTAE intervention was safe and that the study procedures and delivery of the intervention overall were feasible. However, minor amendments to the study protocol were made prior to the commencement of the RCT.

Based on the feasibility study, the recruitment process was considered satisfactory for the planned RCT with a consent rate of 72% which was well above the success criteria recommending a consent rate of > 50%. Furthermore, the drop-out rate of 18% was acceptable as we consider attrition rates between 15–20% as a low risk of bias [50, 51].

Completion time on the questionnaires was acceptable as 92% of the patients answered within 40 min with less than 10% missing items. However, because some patients reported higher symptom intensity after completing the outcome measures, the QOLIBRI was replaced with the shorter version QOLIBRI-OS [45] to reduce the patient burden. Furthermore, the DHI was removed because only a few patients experienced dizziness and this questionnaire was not suitable as it assumes that the respondents have dizziness [52]. Moreover, dizziness is assessed with a question on the RPQ [36] and registered under the BCTT if present.

Because patients may experience increase in several symptoms during the BCTT, we determined to use a pooled-symptom NRS score in the RCT to simplify the scoring process. Due to cardiovascular risk [30] and risks of symptom exacerbation after BCTT we changed the BCTT stop criteria from voluntary exhaustion to 90% of estimated max HR or ≥ 18 Borg RPE. This will probably result in more patients being excluded due to a normal BCTT; however, inactive patients will not be tested towards maximum intensity, and thus, feel safer and the test less provocative [30].

Adherence to the intervention arm in the feasibility study was satisfactory with over 90% attendance rate of SSTAE and BCTT follow-ups. The prescribed home exercises were performed by 75%, and almost all maintained the exercise diary. For the full-scale RCT, we added the Problematic Experience of Therapy Scale (PETS) that will enable us to identify factors that may influence non-adherence [46].

Results from the primary and secondary outcomes were satisfactory in that they showed that most patients in the feasibility study reported reduced symptom burden and were able to exercise at a higher intensity level after 12-weeks of SSTAE. This is in line with a former study that also found that SSTAE contributed to decreased symptom burden, increased exercise tolerance, increased physical activity, and increased health-related quality of life [53] and thus represents a promising option for treatment of persistent PPCS.

A strength of the feasibility study was that the aspects of feasibility were assessed based on specific predefined criteria, which is in line with guidelines [54, 55]. However, the recruitment period was limited to two months and included a small convenience sample. Thus, the generalizability of the feasibility study might be limited. On the other hand, we tested 8% of the sample size required in the RCT which is considered sufficient data to indicate necessary amendments [56]. Furthermore, the findings should be interpreted with caution due to a lack of a control group. However, the purpose was not to determine the effect of the intervention, but to assess its feasibility.

### Trial status

Recruitment for the RCT began in March 2022 and will continue until target sample size has been reached, estimated by the end of 2023.

## Supplementary Information

**Additional file 1: Supplementary file 1.** Feasibility study: Analysis of primary and secondary endpoints.

## Data Availability

The datasets generated and analyzed during the current study are not publicly available due to restrictions from the regional ethical committee but could be made available upon requests through the principal investigator (Ingerid Kleffelgård, inff@live.no).

## Abbreviations

mTBI: Mild Traumatic Brain Injury
PPCS: Post-Concussion Symptoms
BCTT: Buffalo Concussion Treadmill Test
SSTAE: Sub-Symptom Threshold Aerobic Exercise
PROM: Patient Reported Outcome Measures
RPQ: Rivermead Post Concussion Symptoms Questionnaire
GCS: Glasgow Coma Scale
HR: Heart Rate
RCT: Randomized controlled trial
TAU: Treatment as usual
NRS: Numerical Rating Scale
KPH: Kilometer per hour
RPE: Rating of Perceived Exertion
OUH: Oslo University Hospital
DHI: Dizziness Handicap Inventory
PETS: Problematic Experiences of Therapy Scale
