# Supplementary material for: Sub-symptom threshold aerobic exercise for patients with persisting post-concussion symptoms and exercise intolerance after mild traumatic brain injury – a study protocol with a nested feasibility study for a randomized controlled trial
Source: BMC Neurol. 2023 May 3;23:179. doi: 10.1186/s12883-023-03221-7 (PMC10155435; doi:10.1186/s12883-023-03221-7)
Supplement: Supplementary file 1 — Additional file 1: Supplementary file 1. Feasibility study: Analysis of primary and secondary endpoints. [file 12883_2023_3221_MOESM1_ESM.docx]

**Supplementary file 1**

**Feasibility study: Analysis of primary and secondary endpoints**

Eight of the nine patients that completed the SSTAE intervention reported reduced symptom burden on the RPQ, and for six of these patients it was reduced more than the minimal clinical important difference of 4.6 points. The mean change was a reduction of 8.8 (95% CI: 2.93 – 14.63) points in the RPQ from 25.9 (SD 8.7) at baseline to 17.1 (SD 6.7) at follow-up (p< 0.01). On the BCTT, the nine patients that completed the follow-up, increased their HR from mean 83% (SD 13) to 91% (SD 7) of estimated HRmax (p< 0.05) i.e., a change of median 6.9 % (95% CI: 2 – 13.5). (Figure 3). Thus, patients achieved increased capacity to exercise without symptoms or with less symptoms.

Figure 3: Boxplot of change of the primary (A) and secondary (B) outcomes for the upcoming randomised controlled trial, from baseline to 12-weeks results during the feasibility study.


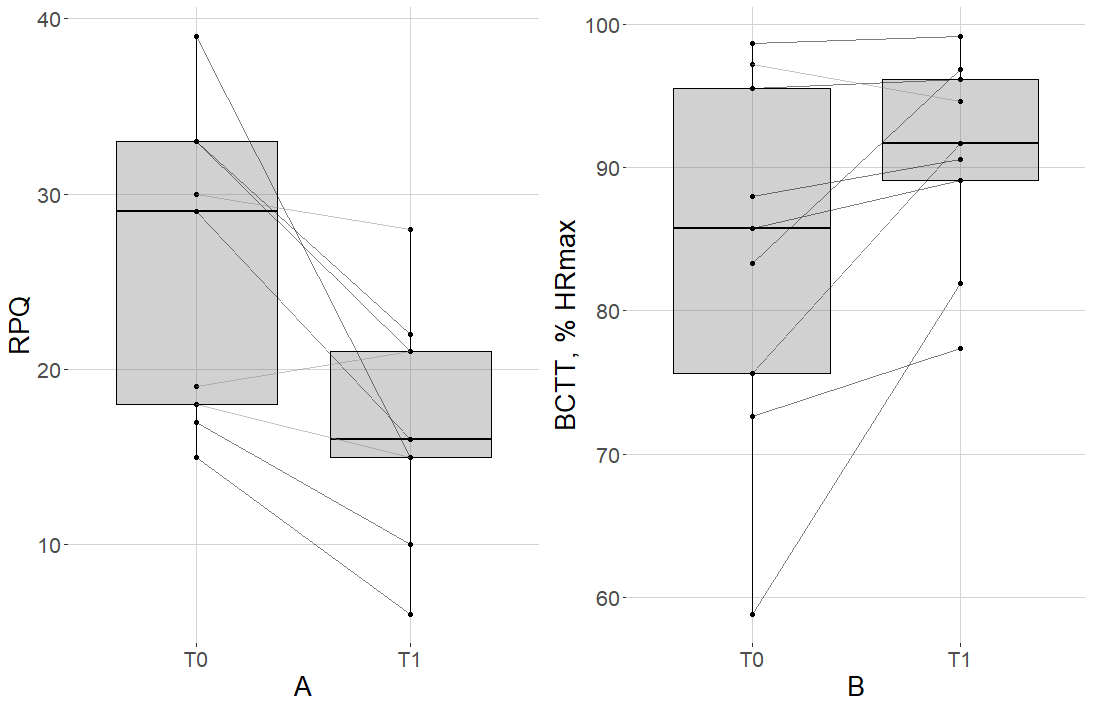


Abbreviations: RPQ, Rivermead Post-Concussion Symptom Questionnaire; BCTT, Buffalo Concussion Treadmill Test. T0 = baseline, T1 = 12 weeks.
